# Modeling APOE ε4 familial Alzheimer’s disease in directly converted 3D brain organoids

**DOI:** 10.3389/fnagi.2024.1435445

**Published:** 2024-08-09

**Authors:** Yunkyung Kim, Hongwon Kim, Byounggook Cho, Saemin An, Soi Kang, Sumin Kim, Jongpil Kim

**Affiliations:** ^1^Department of Chemistry, Dongguk University, Seoul, Republic of Korea; ^2^Department of Chemistry and Chemical Biology, Rutgers, The State University of New Jersey, Piscataway, NJ, United States

**Keywords:** Alzheimer’s disease, direct conversion, 3D modeling, apolipoprotein E, amyloid-beta

## Abstract

Brain organoids have become a valuable tool for studying human brain development, disease modeling, and drug testing. However, generating brain organoids with mature neurons is time-intensive and often incomplete, limiting their utility in studying age-related neurodegenerative diseases such as Alzheimer’s disease (AD). Here, we report the generation of 3D brain organoids from human fibroblasts through direct reprogramming, with simplicity, efficiency, and reduced variability. We also demonstrate that induced brain organoids from APOE ε4 AD patient fibroblasts capture some disease-specific features and pathologies associated with APOE ε4 AD. Moreover, APOE ε4-induced brain organoids with mutant APP overexpression faithfully recapitulate the acceleration of AD-related pathologies, providing a more physiologically relevant and patient-specific model of familial AD. Importantly, transcriptome analysis reveals that gene sets specific to APOE ε4 patient-induced brain organoids are highly similar to those of APOE ε4 post-mortem AD brains. Overall, induced brain organoids from direct reprogramming offer a promising approach for more efficient and controlled studies of neurodegenerative disease modeling.

## Introduction

Brain organoids are three-dimensional cellular structures derived from stem cells that mimic certain features of the human brain. These mini-brain structures have become a valuable tool for studying human brain development, disease modeling, and drug testing (Qian et al., 2019; Chiaradia and Lancaster, 2020). Because they recapitulate some aspects of the human brain’s architecture and functionality, they provide a more physiologically relevant model compared to traditional two-dimensional cell cultures or animal models (Qian et al., 2019). Numerous reports in the literature also support the notion that 3D systems are more physiologically relevant than 2D systems for modeling various diseases (Centeno et al., 2018; Ho et al., 2018; Sacchetto et al., 2020). This enhanced physiological relevance of 3D models is particularly important for diseases where the intricate 3D network of diverse cell types plays a pivotal role in disease progression and pathology. However, despite their potential, the generation of these miniature 3D structures presents several challenges and limitations. One of the major difficulties lies in the time-intensive nature of organoid development, which poses a significant hurdle (Chen et al., 2019). It often takes weeks to months for brain organoids to reach a sufficient level of maturity. Furthermore, inherent heterogeneity within brain organoids adds another layer of complexity (Faravelli et al., 2020). Variability in size, morphology, and cellular composition between batches, and even within the same batch, can impact the reproducibility and reliability of experimental results. Particularly, modeling age-related neurodegenerative diseases like Alzheimer’s with brain organoids faces hurdles including incomplete aging phenotypes in culture, absence of epigenetic phenotypes presents in the aged tissues, and inconsistency within brain organoids (Faravelli et al., 2020; Mertens et al., 2021). In order to address these challenges, in this study, we prepared 3D brain organoids by direct lineage reprogramming, with simplicity, efficiency, and reduced variability.

Alzheimer’s disease (AD) is a progressive neurodegenerative disorder characterized by cognitive decline and memory loss (Jahn, 2013). The pathological hallmarks of AD include the accumulation of amyloid-beta (Aβ) plaques and tau protein tangles in the brain, which lead to neuronal dysfunction and eventual cell death (Serrano-Pozo et al., 2011). Notably, the ε4 allele of the apolipoprotein E gene (APOE ε4) is strongly correlated with fibrillary Aβ burden and Aβ aggregation in late-onset AD, driving amyloid pathology and dystrophic neurites in murine models and human neurons (Huynh et al., 2017; Kim et al., 2022). However, most studies with human primarily rely on 2D culture systems, hampering the development of faithful models that recapitulate the complexity of the human brain in AD and hindering a better understand disease progression in the brains of AD patients. Thus, our study addresses these limitations by presenting a novel approach to generate 3D brain organoids directly from human fibroblasts through the process of direct lineage reprogramming. There are several advantages to modeling AD using 3D organoids derived from patient fibroblasts through direct lineage reprogramming. First, this approach can offer an efficient and precise method for generating aged brain organoids to model dementia. Additionally, direct conversion minimizes variability among organoids, improving reproducibility and robustness of experimental results. Thus, nevertheless, these patient-specific organoids hold significant translational potential for drug screening and therapeutic testing, promising accelerated development of treatments for dementia. Furthermore, our findings reveal that APOE ε4-induced brain organoids with mutant APP overexpression accurately recapitulate the acceleration of AD-related pathologies, offering a more physiologically relevant and patient-specific model of AD. Transcriptome analysis highlights the striking similarity between gene sets specific to APOE ε4 patient-induced brain organoids and those of APOE ε4 post-mortem AD brains, underscoring the translational potential of our approach. Collectively, these results suggest the effectiveness of the 3D human induced brain organoids culture system for accurate modeling of AD, indicating its expanded application for studying progression of other neurodegenerative diseases. However, this model predominantly features glutamatergic and GABAergic neurons, which may limit the analysis of contributions from glial cells and other cell types that are important for fully understanding AD pathology.

Overall, this study demonstrates the promise of induced brain organoids derived through direct reprogramming as a more efficient and controlled platform for studying neurodegenerative diseases, particularly AD. This approach opens new avenues for unraveling the complexities of AD pathogenesis and accelerating the development of novel therapeutics.

## Materials and methods

### Culture of human fibroblasts

The human fibroblasts were cultured in a medium consisting of DMEM supplemented with 10% fetal bovine serum, 1% nonessential amino acids (Gibco, Waltham, MA, USA), 0.1% β-mercaptoethanol (Gibco), and 1% penicillin/streptomycin (Gibco). Human fibroblasts from both healthy individuals (GM23967, male, APOE ε3/3 genotype; AG21158, female, APOE ε2/3 genotype) and AD patients (AG04402, male, APOE ε3/4 genotype; AG05810, female, APOE ε3/4 genotype; AG11414, male, APOE ε3/4 genotype) were purchased from the Coriell Cell Repository (Camden, NJ) (Supplementary Table 1).

### Direct lineage reprogramming of human fibroblasts into induced neurons

Lentiviruses were produced by HEK293T cells transfected with lentiviral constructs (psPAX2 and pMD2.G), and with each construct containing direct conversion factors into neurons (Ascl1, Brn2, Myt1l, or NeuroD1) using calcium phosphate co-precipitation. The culture medium was replaced 24 h after transfection, and the viruses were harvested 72 h later following a previously published protocol (Kim et al., 2019). Human induced neurons were generated by transducing human fibroblasts with lentiviruses carrying FUW-Ascl1, Brn2, Myt1l, and NeuroD1 three times over 2 days. After 48 to 72 h of infection, the medium was exchanged with N2 medium, consisting of DMEM/F12 supplemented with insulin (25 μg/ml), progesterone (20 nM), transferrin (50 μg/ml), putrescine (100 μM), laminin (1 μg/ml), FGF basic (25 μg/ml), BDNF (10 μg/ml), Forskolin (5 μM), and 1% penicillin/streptomycin.

### Immunofluorescence analysis

2D-cultured induced neurons and 3D-cultured induced brain organoids from Alzheimer’s disease-patients were fixed in 4% paraformaldehyde (Sigma). Following fixation, induced brain organoids were placed in an embedding mold containing Optimal Cutting Temperature (OCT) compound, stored at −70°C, and then cut into cryosections. Slides of fixed induced neurons and cryosections of induced brain organoids were washed twice with 1× PBS containing 0.1% Triton-X (PBST). After blocking with PBST supplemented with 1% BSA for 1 h, the samples were incubated overnight at 4°C with primary antibodies [anti-βIII-tubulin, Sigma-Aldrich, St. Louis, MO; anti-β-Amyloid (6E10), BioLegend, San Diego, CA; anti-phospho-Tau (Ser202, Thr205), Invitrogen, Carlsbad, CA; anti-VGLUT1, Invitrogen; anti-MAP2, Cell Signaling, Danvers, MA; Synapsin 1, Invitrogen; anti-PSD-95, Invitrogen; anti-GAD-65/67, Santa Cruz, Dallas, TX]. Following subsequent washing with PBST, the samples were incubated with appropriate secondary antibodies at RT for 2 h, and counterstained with DAPI. Representative images were obtained using a Zeiss confocal microscope (Zeiss, Oberkochen, Germany, LSM800). Fluorescence intensity was measured using a confocal microscope and normalized to background levels. Random counting areas were selected in each sample to ensure unbiased visualization. An investigator blinded to the experimental conditions conducted all analyses. Quantification of analyses was performed on entire organoids to ensure comprehensive data collection. Image J software was used for particle analysis and quantification of immunofluorescent signals within regions of interest. These procedures were carried out consistently on the same confocal microscope with identical settings.

### Thioflavin T staining

To stain cryosections of 3D induced brain organoids directly reprogrammed from either healthy donors or AD patients with Thioflavin T (ThT; Sigma-Aldrich), ThT was initially dissolved in ddH_2_O to a concentration of 2 mM and then diluted 200-fold in 50% ethanol to achieve a final concentration of 10 μM. Samples were subsequently incubated with 10 μM ThT for 15 min, followed by two 5-min washes with 50% ethanol, and then washed with PBST.

### RNA isolation and quantitative RT-PCR analysis

The RNA extraction from 2D-cultured induced neurons or 3D-cultured induced brain organoids was carried out using the eCube Tissue RNA Mini Kit (Philekorea, Seoul, Korea) following the manufacturer’s protocol. Briefly, 1 μg of the isolated RNA was reverse-transcribed using the AccuPower^®^ CycleScript RT PreMix (Bioneer, Daejeon, Korea). For subsequent qRT-PCR analysis, as described previously (Kim et al., 2021), we used the Rotor-Gene Q real-time PCR cycler (Qiagen, Hilden, Germany) with appropriate primer sets and AccuPower^®^ PCR PreMix (Bioneer, Daejeon, Korea). Quantitative PCR was performed to assess gene expression levels, with all reactions carried out in triplicate. Gene expression of each marker was normalized against GAPDH in each sample.

### Western blot analysis

For Western blot analysis, samples of 3D induced brain organoids with APOE ε3 or ε4 were washed with 1× PBS and lysed using RIPA buffer (1% NP-40, 0.5% DOC, 0.1% SDS, and 150 mmol/L NaCl in 50 mmol/L Tris, pH 8.0; Sigma-Aldrich) supplemented with 1× proteinase inhibitor mixture (GenDepot, Barker, TX). Extracted proteins were quantified using the Pierce™ Bradford Protein Assay Kit according to the manufacturer’s instructions (Thermo Fisher Scientific, Waltham, MA). Following a previously described protocol (Park et al., 2019), 10 μg of each sample was mixed with 5× loading dye and boiled at 100°C for 10 min. Subsequently, proteins were electrophoresed on a 12% sodium dodecyl sulfate-polyacrylamide gel and then transferred to a nitrocellulose membrane (GE Healthcare, Piscataway, NJ, USA). After blocking with TBS containing 0.1% Tween-20 and 5% BSA for 1 h, the membranes were incubated overnight at 4°C with primary antibodies [anti-β-Amyloid (12F4), BioLegend; anti-β-actin, Santa Cruz]. Following incubation, membranes were washed with TBST and then incubated with suitable HRP-conjugated secondary antibodies for 2 h at room temperature. The resulting bands were visualized using an ECL kit (Dogen, Seoul, Korea) and representative images were obtained using Chemidoc TRS + (Bio-Rad Laboratories, Hercules, CA, USA). Western blot bands were quantified using Image Lab Software, and protein levels were normalized to β-actin.

### Bulk RNA sequencing and analysis

For bulk RNA sequencing, total RNA from 3D brain organoids was extracted on day 20. RNA integrity was assessed before library preparation using the TruSeq Stranded mRNA Sample Prep Kit was used according to the manufacturer’s instructions (Illumina, San Diego, CA, USA). The libraries were sequenced in a paired-end fashion with 100 bp reads on the Illumina HiSeq 2500 platform. Alignment was carried out using STAR, while raw counts were generated utilizing bowtie2. Subsequently, variance stabilizing transformation normalization (vst) and differential expression analyses were conducted using DESeq2. For gene set enrichment analysis (GSEA), GO Biological Processes, GO Cellular Components, and KEGG pathways were examined via DAVID, with redundancy-trimmed GSEA plots visualized using REVIGO.

### Statistical analysis

All data are presented as mean ± SEM of each independent experiments, with “n” representing the number of individual experiments. Particularly, the N value in our analyses represents the number of organoids analyzed per patient. Statistical tests were conducted per patient to ensure the validity and accuracy of our results. Each experiment included at least three independent technical replicates. Statistical analysis was performed using GraphPad Prism, which involved analysis of variance (ANOVA) followed by Tukey–Kramer multiple comparison tests to assess significant intergroup differences. Group differences were considered statistically significant at *P < 0.05, **P < 0.01, ***P < 0.001, and ****P < 0.0001. Detailed statistical methods are provided in the figure legends.

## Results

### Generation of 3D brain organoids directly induced from human fibroblasts

**FIGURE 1:**
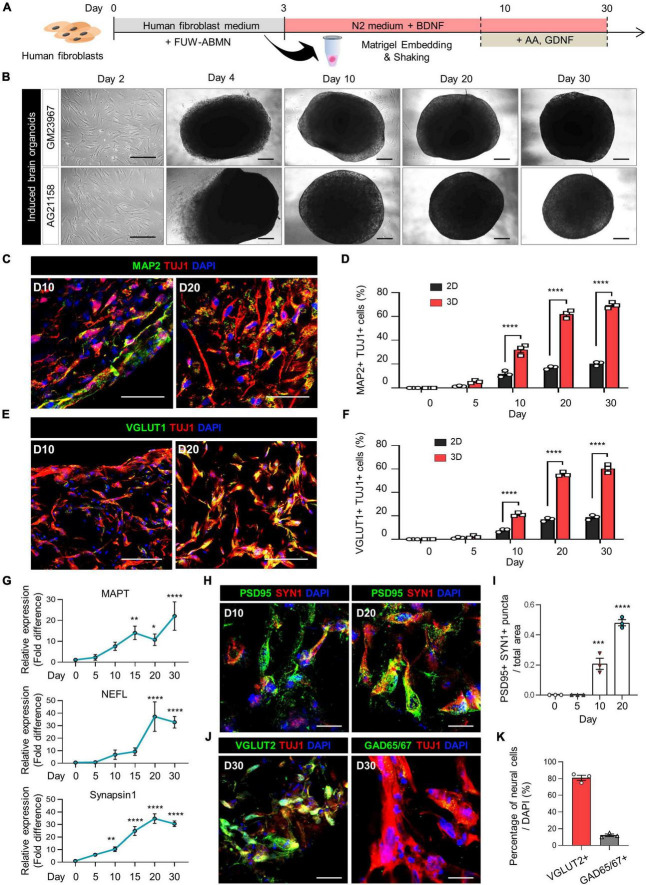
Generation of induced brain organoids from human fibroblasts using 3D direct reprogramming. **(A)** Schematic diagram illustrating the generation of directly reprogrammed 3D-cultured induced brain organoids from human fibroblasts. **(B)** Bright-field images of 2D-cultured human fibroblasts at day 2 and 3D-cultured induced brain organoids at days 4, 10, 20, and 30 after seeding. Scale bar = 500 μm. **(C)** Immunostaining of Tuj1+Map2+ cells in 3D induced brain organoids derived from GM23967 at different time points. Scale bar = 100 μm. **(D)** Quantification of Tuj1- and Map2-positive cells in 2D-cultured plates and 3D-cultured induced brain organoids at different time points. Data represent mean ± SEM. Two-way ANOVA with Tukey’s multiple comparisons test, ****P < 0.0001, *n* = 3 per fibroblast. **(E)** Immunostaining of Tuj1+Vglut1+ cells in 3D induced brain organoids derived from GM23967 at different time points. Scale bar = 100 μm. **(F)** Quantification of Tuj1- and Vglut1-positive cells in 2D-cultured plates and 3D-cultured induced brain organoids at different time points. Data represent mean ± SEM. Two-way ANOVA with Tukey’s multiple comparisons test, ****P < 0.0001, *n* = 3 per fibroblast. **(G)** Quantitative RT-qPCR analysis of 3D induced brain organoids regarding MAPT and NEFL (neuronal markers), as well as Synapsin1 (a pre-synaptic marker), at different time points. Data represent mean ± SEM. One-way ANOVA with Tukey’s multiple comparisons test, *P < 0.05, **P < 0.01, ***P < 0.001, ****P < 0.0001, *n* = 3 per sample. **(H)** Immunofluorescence staining of Syn1- and PSD95-positive cells in 3D induced brain organoids derived from GM23967. Scale bar = 20 μm. **(I)** The number of Syn+PSD95+ puncta per total area at different time points. Data represent mean ± SEM. Ordinary one-way ANOVA with Dunnett’s multiple comparisons test, ***P < 0.001, ****P < 0.0001, *n* = 3 per fibroblast. **(J)** Immunofluorescence staining of Tuj1+Vglut2+ or Tuj1+GAD65/67+ cells in 3D induced brain organoids derived from GM23967 at day 30. Scale bar = 20 μm. **(K)** Percentage of Vglut2- or GAD65/67-positive neural cells. *n* = 3 per fibroblast.

Previous studies have demonstrated that post-natal human fibroblasts can be directly reprogrammed into functional neurons through the expression of Ascl1, Brn2, and Myt1l supplemented with Neurod1/2 (ABMN) (Vierbuchen et al., 2010; Pang et al., 2011). Thus, to generate 3D-cultured induced brain organoids through direct lineage reprogramming, human fibroblasts were transduced with lentiviruses constitutively expressing ABMN and then embedded in Matrigel to form 3D brain organoid structures. Then, these induced brain organoids were maintained using a shaker in a CO2 incubator until day 30 (Figure 1A). We observed that the Matrigel-embedded directly converted cells efficiently condensed and formed a globular shape, maintaining a constant size of about 2.5 mm over time, as visualized by bright-field microscopy (Figure 1B).

**FIGURE 2:**
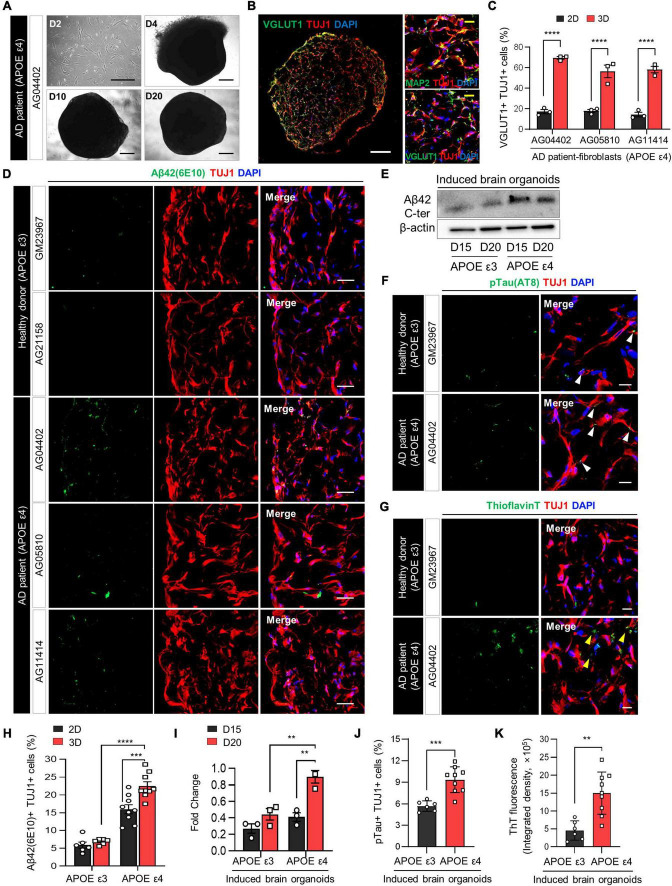
Increase in AD-related phenotypes in APOE ε4-expressing 3D induced brain organoids from AD patients compared to those expressing APOE ε3. **(A)** Bright-field images of 2D-cultured fibroblasts from an AD patient (AG04402) at day 2 and 3D-cultured induced brain organoids at days 4, 10, and 20. Scale bar = 500 μm. **(B)** Representative immunofluorescence images of Tuj1+Map2+ or Tuj1+Vglut1+ cells in 3D induced brain organoids derived from AG04402 (APOE ε4) at day 20. Scale bar = 250 μm (white) or 20 μm (yellow). **(C)** Quantification of Tuj1- and Vglut1-positive cells in 3D induced brain organoids derived from AD patients at day 20. Data represent mean ± SEM. Two-way ANOVA with Tukey’s multiple comparisons test, ****P < 0.0001, *n* = 3 per fibroblast. **(D)** Immunostaining of Aβ42(6E10)- and Tuj1-positive cells in 3D induced brain organoids from healthy donors (GM23967 and AG21158) and AD patients (AG04402, AG05810, and AG11414) at day 20. Scale bar = 50 μm. **(E)** Western blot analysis of β-Amyloid in APOE ε3- or APOE ε4-expressing 3D induced brain organoids on days 15 and 20. **(F)** Representative immunofluorescence images of phosphorylated tau (pTau) in 3D induced brain organoids from a healthy donor or an AD patient at day 20. White arrows indicate areas that are double positive for pTau and Tuj1. Scale bar = 20 μm. **(G)** Representative images of Thioflavin T (ThT) staining in 3D induced brain organoids from a healthy donor or an AD patient at day 20. Yellow arrows indicate areas that are ThT-positive and correspond to the extracellular region. Scale bar = 20 μm. **(H)** Quantification of Tuj1- and Aβ42(6E10)-positive cells in 3D induced brain organoids derived from APOE ε3- or APOE ε4-fibroblasts. Data represent mean ± SEM. Two-way ANOVA with Tukey’s multiple comparisons test, ***P < 0.001, ****P < 0.0001, *n* = 3 per fibroblast for both APOE ε3 (GM23967 and AG21158) and APOE ε4 (AG04402, AG05810, and AG11414). **(I)** Quantification of Western blot analysis data for β-Amyloid in APOE ε3- or APOE ε4-expressing 3D induced brain organoids on days 15 and 20. Data represent the mean ± SEM. Two-way ANOVA with Tukey’s multiple comparison test; **P < 0.01, *n* = 3 per sample. **(J)** Quantification of pTau- and Tuj1-positive cells in 3D induced brain organoids derived from APOE ε3 or APOE ε4 fibroblasts. Data represent mean ± SEM. Unpaired *t*-test, ***P < 0.001. *n* = 3 per fibroblast for both APOE ε3 (GM23967 and AG21158) and APOE ε4 (AG04402, AG05810, and AG11414). **(K)** Measurement of ThT fluorescence as integrated density values using ImageJ software in 3D induced brain organoids from APOE ε3 or ε4 fibroblasts. Data represent mean ± SEM. Unpaired *t*-test, **P < 0.01. *n* = 3 per fibroblast for both APOE ε3 (GM23967 and AG21158) and APOE ε4 (AG04402, AG05810, and AG11414).

The expression of mature neuronal markers, including Tuj1, Map2, and Vglut1, was confirmed in these induced brain organoids (Figures 1C, E). Interestingly, the number of Tuj1+Map2+ cells gradually increased over time, with higher counts observed in 3D induced brain organoids compared to those in 2D culture at each time point (Figure 1D). Specifically, the proportion of both Tuj1+Map2+ cells and Tuj1+Vglut1+ cells in induced brain organoids exceeded 50% after day 20 (Figures 1D, F). Additionally, we observed a remarkable increase in gene expression for neuronal markers MAPT and NEFL, along with the presynaptic marker Synapsin1 (SYN1) in these induced brain organoids over the entire culture period (Figure 1G). Furthermore, immunofluorescence staining for SYN1 and PSD95, the pre- and post-synaptic markers, was conducted to evaluate the synaptic development of induced brain organoids. SYN1+PSD95+ puncta per total area were detected after day 10, and nearly doubled by day 20 (Figures 1H, I). Moreover, we observed that the percentage of Vglut2+ cells exceeded 80%, indicating that the majority of neural cells in 3D-cultured induced brain organoids have differentiated into glutamatergic neurons within 30 days (Figures 1J, K). We confirmed that our organoids maintain viability beyond 30 days (Supplementary Figures 1C, F), indicating their potential for extended application in long-term studies. Furthermore, while previous studies have shown that iPSC-derived cerebral organoids produce a diverse range of cell types (Vitale et al., 2012; Velasco et al., 2019), highlighting inherent variability in cell type representation within these structures, we observed reduced cellular variability with lower standard deviations in Vglut1+ cell proportions across multiple organoids (Figures 1F, 2C and Supplementary Figure 2F). Additionally, our 3D brain organoids showed an absence of glial populations (Supplementary Figures 1D, E, G, H). Taken together, these results demonstrate that directly reprogrammed 3D induced brain organoids from human fibroblasts exhibit well-developed characteristics of mature neurons, including synapse formation.

### Modeling APOE ε4 AD in 3D induced brain organoids

The APOE ε4 allele, widely recognized as the primary risk factor for Alzheimer’s disease (AD), induces broad molecular and cellular changes linked to AD phenotypes, prominently enhancing amyloid-beta (Aβ) deposition in the brain (Lin et al., 2018). To demonstrate whether directly reprogrammed brain organoids can efficiently recapitulate neurodegenerative diseases such as Alzheimer’s disease, we initially generated 3D induced brain organoids derived from APOE ε4-expressing AD patient fibroblasts with a familial origin. Subsequently, we examined the effect of APOE ε4 on AD-related pathogenesis within the 3D environment of induced brain organoids. Notably, induced brain organoids from fibroblasts of AD patients with APOE ε4 exhibited the development of well-defined globular 3D structures over time (Figure 2A and Supplementary Figure 1A). Consistent with previous results, at a day 20, we observed a significant increase in Tuj1+Vglut1+ cells within 3D induced brain organoids from APOE ε4 AD patients, compared to 2D-cultured induced neurons (Figures 2B, C and Supplementary Figure 1B).

The accumulation of amyloid Aβ in the brain is one of the most significant pathological features of AD (Gu and Guo, 2013). Therefore, we also compared the Aβ aggregation in induced brain organoids derived from APOE ε4-expressing AD patients. The number of Aβ42+Tuj1+ cells was significantly increased in induced brain organoids expressing APOE ε4, consistent with higher Aβ42 protein levels observed in APOE ε4-induced brain organoids relative to APOE ε3-induced brain organoids by day 20 (Figures 2D, E, H, I). Furthermore, Aβ accumulation in induced neurons from each sample was also increased in the 3D culture environment compared to 2D plate culture, implying the significant role of the 3D structure in accurately mimicking the AD condition (Figure 2H). We also observed elevated phosphorylated tau (pTau) deposition in cell bodies and dendrites, as well as increased Thioflavin T (ThT) positive aggregates in 3D brain organoids harboring APOE ε4 compared to those with APOE ε3 (Figures 2F, G, J, K and Supplementary Figures 2A, B). In particular, the ThT-positive extracellular regions highlighted by yellow arrows exhibited a significant increase in APOE ε4 3D brain organoids, consistent with previous research demonstrating extracellular Aβ deposition in 3D culture (Kim et al., 2015). Although we sought to investigate whether the age of APOE ε4 donors correlates with the degree of pathological features in their induced brain organoids, we did not observe significant differences among the APOE ε4 donors (Supplementary Figures 2C–E). These findings collectively suggest that generation of 3D induced brain organoids exhibits AD-related pathologies associated with APOE ε4 more rapidly and efficiently than conventional 2D culture or iPSC-based methods, thus indicating its suitability for precise AD modeling.

### Acceleration of Aβ-associated pathologies in APOE ε4 3D brain organoids with APP mutation

**FIGURE 3:**
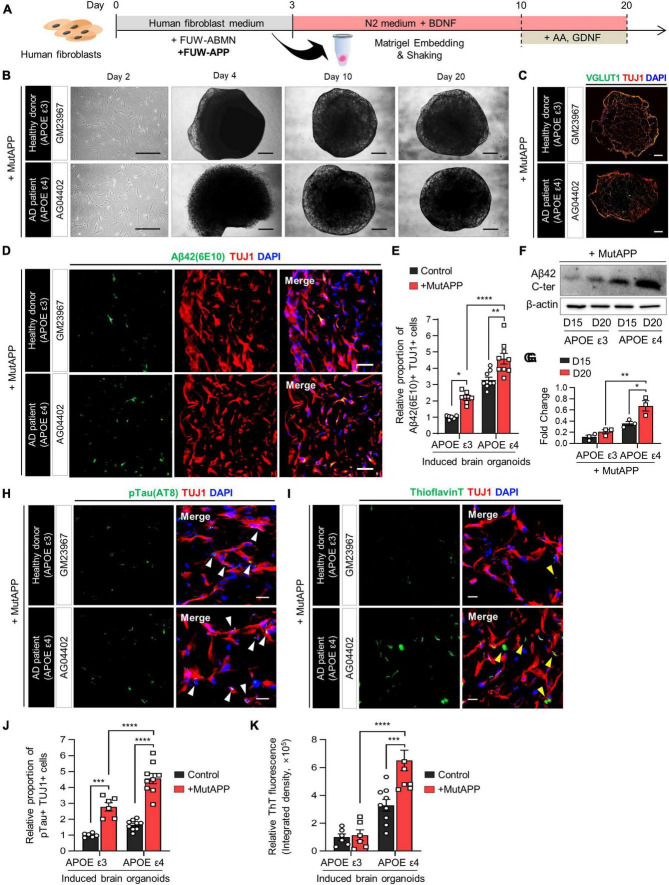
Acceleration of Aβ deposition and Aβ-associated pathologies in APOE ε4 3D induced brain organoids with APP mutation. **(A)** Schematic diagram of the generation of 3D-cultured induced brain organoids with APP mutation from AD patient fibroblasts. **(B)** Bright-field images of 2D-cultured human fibroblasts at day 2 and 3D-cultured induced brain organoids at day 4, 10, and 20 from a healthy donor (GM23967) or an AD patient (AG04402). Scale bar = 500 μm. **(C)** Immunostaining of Tuj1+Vglut1+ cells in induced brain organoids with APP mutation from a healthy donor or an AD patient at day 20. Scale bar = 250 μm. **(D)** Representative immunofluorescence images of Aβ42(6E10)- and Tuj1-positive cells in induced brain organoids with APP mutation from a healthy donor or an AD patient at day 20. Scale bar = 50 μm. **(E)** Quantification of Tuj1- and Aβ42(6E10)-positive cells in induced brain organoids with APP mutation derived from APOE ε3 or APOE ε4 fibroblasts. Data represent mean ± SEM. Unpaired *t*-test, *P < 0.05, **P < 0.01, ****P < 0.0001. *n* = 3 per fibroblast for both APOE ε3 (GM23967 and AG21158) and APOE ε4 (AG04402, AG05810, and AG11414). **(F,G)** Western blot analysis of β-Amyloid in APOE ε3- or APOE ε4-expressing induced brain organoids with APP mutation on day 15 and 20 with quantification. Data represent mean ± SEM. Two-way ANOVA with Tukey’s multiple comparisons test, *P < 0.05, **P < 0.01, *n* = 3 per sample. **(H)** Representative immunofluorescence images of phosphorylated tau (pTau) in induced brain organoids with APP mutation from a healthy donor or an AD patient at day 20. White arrows indicate areas that are double positive for pTau and Tuj1. Scale bar = 20 μm. **(I)** Representative images of Thioflavin T (ThT) staining in induced brain organoids with APP mutation from a healthy donor or an AD patient at day 20. Yellow arrows indicate areas that are ThT-positive and correspond to the extracellular region. Scale bar = 20 μm. **(J)** Quantification of pTau- and Tuj1-positive cells in induced brain organoids with APP mutation derived from APOE ε3 or ε4 fibroblasts. Data represent mean ± SEM. Unpaired *t*-test, ***P < 0.001, ****P < 0.0001. *n* = 3 per fibroblast for both APOE ε3 (GM23967 and AG21158) and APOE ε4 (AG04402, AG05810, and AG11414). **(K)** Measurement of ThT fluorescence as integrated density values using ImageJ software in induced brain organoids with APP mutation from APOE ε3 or ε4 fibroblasts. Data represent mean ± SEM. Unpaired *t*-test, ***P < 0.001, ****P < 0.0001. *n* = 3 per fibroblast for both APOE ε3 (GM23967 and AG21158) and APOE ε4 (AG04402, AG05810, and AG11414).

The acceleration of AD-related pathologies occurs when APOE ε4 increases Aβ production by accelerating APP endocytosis transport mediated by low-density lipoprotein receptor-related protein, thereby initiating and promoting the accumulation, aggregation, and deposition of Aβ in the brain (Pietrzik et al., 2002; Tachibana et al., 2019). Therefore, we examined whether directly reprogrammed 3D brain organoids can effectively model the accumulative effect of amyloid precursor protein (APP) expression with APOE ε4. To prepare APOE ε4-induced brain organoids expressing APP, fibroblasts from AD patients were transduced with lentivirus constitutively expressing mutant APP, alongside ABMN lentiviruses (Figure 3A). After 2 days, transduced human fibroblasts were detached and embedded in Matrigel to generate 3D structures, resulting in a globular shape remained consistent and stable in size after day 10 (Figure 3B). At day 20, the majority of cells in induced brain organoids expressing APP were positive for Tuj1 and Vglut1, verifying the neuronal identity of induced neurons through 3D direct lineage reprogramming (Figure 3C). To investigate potential synaptic deterioration associated with APOE variants, we compared the percentage of cells co-expressing Tuj1 and Vglut1 in induced brain organoids derived from APOE ε4 and APOE ε3 fibroblasts, finding no significant differences (Supplementary Figure 2F).

We found that Aβ42- and Tuj1-positive cells were significantly increased in induced brain organoids with both APOE ε3 and ε4 in conjunction with APP expression (Figures 3D, E). Additionally, the protein level of Aβ42 was elevated in induced brain organoids expressing APOE ε4 and constitutive APP (Figures 3F, G). Moreover, the deposition of pTau, co-localized with Tuj1 in induced brain organoids, was further enhanced by the expression of APP in the presence of APOE ε4 (Figures 3H, J). Also, upon expression of APP, the ThT-positive area overlapped with cell bodies and neurites, exhibiting an increase in both APOE ε3 and ε4-induced brain organoids. Particularly, we observed an increased ThT region corresponding to the extracellular space in APOE ε4-induced brain organoids, as indicated by yellow arrows (Figures 3I, K). These findings suggest that the generation of directly reprogrammed 3D brain organoids effectively reflects the exacerbation of AD pathologies through the co-expression of APOE ε4 and APP.

### Transcriptional dynamics of APOE ε4 3D brain organoids

**FIGURE 4:**
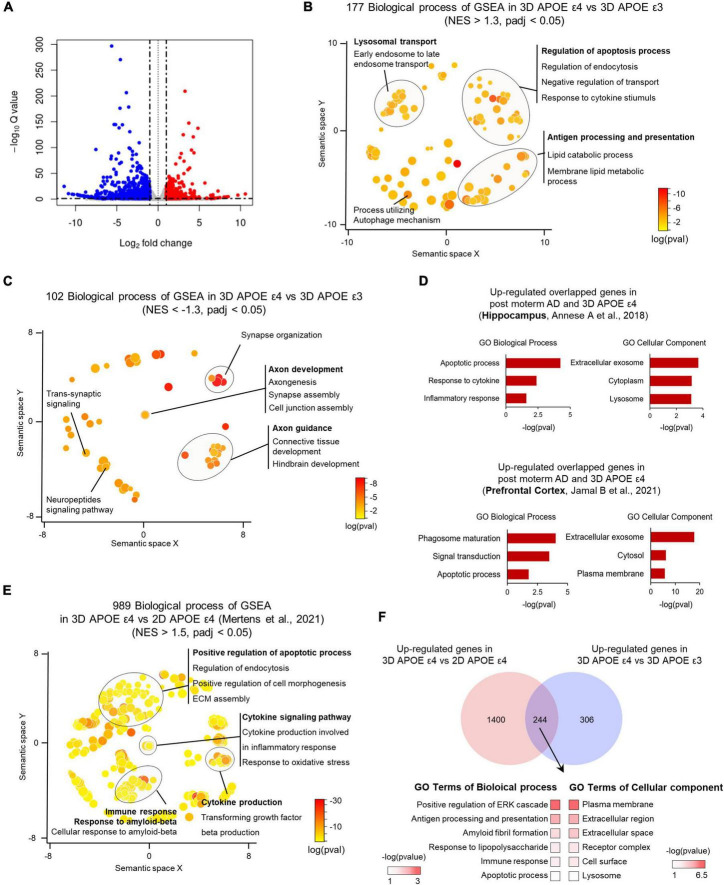
Transcriptome analysis of APOE ε4-expressing 3D induced brain organoids. **(A)** Volcano plot for APOE ε4 versus ε3 DE genes in 3D induced brain organoids. **(B)** Enriched GO terms for genes significantly upregulated in APOE ε4-induced brain organoids versus APOE ε3-induced brain organoids within a redundancy-trimmed semantic space (NES > 1.3, adjusted *p*-value < 0.05). **(C)** Enriched GO terms for genes significantly downregulated in APOE ε4-induced brain organoids versus APOE ε3-induced brain organoids within a redundancy-trimmed semantic space (NES < –1.3, adjusted *p*-value < 0.05). **(D)** Bar graph showing GO categories from overlapping genes that are commonly changed in the same direction in APOE ε4-induced brain organoids and post-mortem brain (hippocampus and prefrontal cortex). **(E)** Enriched GO terms in significantly upregulated 3D APOE ε4-induced brain organoids versus 2D APOE ε4-induced neurons genes in redundancy-trimmed semantic space (NES < 1.5, adjusted *p*-value < 0.05). **(F)** Venn diagram and GO categories showing overlapping up-regulated genes from 3D versus 2D APOE ε4 conditions and from 3D APOE ε4 versus ε3 conditions.

To investigate the molecular alterations associated with AD pathologies influenced by the APOE ε4 allele in 3D-cultured environment, we conducted RNA sequencing of APOE ε4-expressing AD-patient induced brain organoids in comparison to those expressing APOE ε3 allele. RNA-seq data analysis identified several significantly upregulated or downregulated DEGs between APOE ε3 and APOE ε4 patients (Supplementary Table 2). Interestingly, when comparing APOE ε4 with ε3, we identified 1,586 significantly differentially expressed genes (DEGs) within 3D induced brain organoids (Figure 4A and Supplementary Figures 3A, B). Gene set enrichment analysis (GSEA) indicated that 177 categories were enriched in the most significantly (padj < 0.05) upregulated genes of APOE ε4-induced brain organoids, covering a semantic space around lysosomal transport, and apoptosis process. In contrast, 102 categories that are downregulated genes showed axon development, axon guidance and synapse organization (Figures 4B, C). We next examined whether the genes found in 3D APOE ε4-induced brain organoids are consistent with those identified in transcriptome database of APOE ε4 post-mortem brain from hippocampus (HP) and prefrontal cortex (PFC). First, 230 genes were obtained in comparison with hippocampus and 3D APOE ε4 organoid, and 210 overlapping genes were acquired from prefrontal cortex. Interestingly, a similar number of overlapping genes were identified when comparing 3D APOE ε4 organoids with two regions of human AD brain, and the gene ontology analysis confirmed that their biological features including apoptosis, exosome also similarly represented (Figure 4D and Supplementary Figure 4A). Conversely, 3D APOE ε4-induced brain organoids consistently exhibited the highest overlap with down-regulated categories related to synapse and axon, which are commonly observed in both systems (Supplementary Figure 4B). These data suggest that representative traits of AD, such as impaired neuronal function, apoptosis, and inflammatory response, are reflected in 3D APOE ε4-induced brain organoids. Moreover, to further determine whether 3D culture environments can contribute to deepening AD pathology compared to 2D culture, we conducted differential expression gene analysis with published 2D APOE ε4-induced neurons data (Mertens et al., 2021) (Supplementary Figure 5). We found that highly enriched GSEA results showing AD traits, such as cytokine-mediated signaling pathway, oxidative stress and response to Aβ in 3D environments. Additionally, we found that the gene set linked to extracellular matrix (ECM) assembly and cell morphogenesis is more prominent in 3D environments compared to 2D cultures (Figure 4E). Interestingly, we discovered 224 shared genes between DEGs in 3D organoids with different APOE genotypes (APOE ε4 versus APOE ε3) and those from both 2D and 3D culture environments (3D versus 2D) (Figure 4F). These genes are relate to plasma membrane, extracellular regions, and amyloid fibril formation. These results suggest that 3D organoid modeling overcomes the limitations of the lack of cell-to-cell interactions in 2D culture, and builds a deeper AD pathological phenotype through the construction of their surrounding ECM. In summary, using 3D brain organoids generated through direct lineage reprogramming for AD modeling will provide a powerful tool for advancing our understanding of the disease and developing new treatment strategies.

## Discussion

In this study, we report a novel approach to modeling APOE ε4 Alzheimer’s disease (AD) using 3D induced brain organoids directly converted from AD patient fibroblasts. Brain organoids have emerged as a valuable tool for studying various aspects of human brain development, disease pathology, and drug testing (Chiaradia and Lancaster, 2020). However, conventional methods for generating brain organoids from pluripotent stem cells often suffer from limitations such as time intensity and incomplete maturation of neurons, which restrict their utility, especially in studying age-related neurodegenerative diseases like AD (Mertens et al., 2021). We demonstrate that inducing brain organoids from human fibroblasts through direct conversion offers several advantages, including simplicity, efficiency, and reduced variability compared to traditional methods of generating brain organoids.

Importantly, we show that brain organoids derived from fibroblasts of patients with APOE ε4 allele, a major genetic risk factor for AD, capture disease-specific features and pathologies associated with APOE ε4 AD. When modeling APOE ε4 AD through direct reprogramming of aged cells, the most significant advantage is the creation of induced brain organoids that retain the epigenetic alterations related to cellular aging (Mertens et al., 2021). However, brain organoids produced from pluripotent stem cells undergo epigenetic resetting, potentially hindering their ability to accurately replicate the epigenetic modifications linked to aging. Considering these aspects, the generation of brain organoids via direct reprogramming and subsequent disease modeling enables a more accurate portrayal of the disease’s condition. Consistently, by introducing mutant amyloid precursor protein (APP) into the APOE ε4-induced brain organoid, we were able to faithfully recapitulate the acceleration of AD-related pathologies, suggesting that this model provides a physiologically relevant and patient-specific representation of AD. Transcriptome analysis further supports the validity of this model, revealing that gene sets specific to APOE ε4 patient-induced brain organoids closely resemble those found in APOE ε4 post-mortem AD brains. This finding underscores the potential of induced brain organoids from direct reprogramming as a promising approach for studying neurodegenerative diseases with increased efficiency and control.

However, there are still several potential challenges and limitations when generating brain organoids through direct reprogramming. For examples, while direct reprogramming offers simplicity and efficiency, direct reprogramming may result in the generation of a heterogeneous population of cells within the organoids, including various neural cell types and potentially non-neural cells. This heterogeneity could affect the reproducibility and consistency of disease phenotypes within the brain organoids. Moreover, induced brain organoids derived from direct reprogramming may lack the intricate neuronal circuitry and functional connectivity observed in other iPSC-derived brain organoids. To overcome these challenges, we could enhance direct reprogramming by incorporating additional factors that promote more comprehensive neuronal differentiation and maturation. Moreover, additional culture systems, such as co-culturing with supporting cell types could also be employed to promote the development of mature neuronal phenotypes and functional connectivity in the organoids. These approaches could improve the suitability of directly reprogrammed brain organoids for studying adult-onset neurodegenerative diseases. Moreover, while our model effectively captures the contribution of amyloid β production and deposition, it is well known that this represents only one aspect of the complex pathology of AD. Therefore, future studies should incorporate additional elements of AD pathology, such as tau protein tangles and neuroinflammation, to provide a more comprehensive understanding of the disease. Finally, in the transcriptome analysis, the changes seen in neuronal characteristics are important and may provide some novel therapeutic targets. However, these findings need to be further examined in a system that includes glial and other cell types to fully understand their therapeutic potential and relevance to the complex cellular environment of the human brain.

Overall, our study highlights the utility of 3D brain organoids generated from direct reprogramming in modeling AD and provides insights into the pathological mechanisms underlying the disease. The ability to recapitulate disease-specific features and pathologies in a patient-specific manner suggests that this model could facilitate drug screening and development of personalized therapeutic strategies for AD.

## Data availability statement

The authors declare that data supporting the findings of this study are available within the article and its Supplementary Information file or from the corresponding author on request. The bulk RNA-seq datasets generated in this study have been deposited in the SRA accession numbers (SRR29673391, SRR29673392, SRR29673393, SRR29673394). Public database of bulk RNA-seq utilized from ArrayExpress: E-MTAB-10352.

## Ethics statement

Ethical approval was not required for the studies on humans in accordance with the local legislation and institutional requirements because only commercially available established cell lines were used.

## Authors contributions

YK: Validation, Formal analysis, Data curation, Visualization, Investigation, Writing – original draft, Writing – review & editing. HK: Conceptualization, Validation, Formal analysis, Data curation, Visualization, Investigation, Writing – original draft, Writing – review & editing. BC: Validation, Formal analysis, Data curation, Visualization, Investigation, Writing – original draft, Writing – review & editing. SA: Investigation, Writing – original draft. SoK: Investigation, Writing – original draft. SuK: Investigation, Writing – original draft. JK: Conceptualization, Validation, Writing – original draft, Writing – review & editing, Funding acquisition, Supervision, Project administration.
