# Supplementary material for: Modeling APOE ε4 familial Alzheimer’s disease in directly converted 3D brain organoids
Source: Front Aging Neurosci. 2024 Aug 9;16:1435445. doi: 10.3389/fnagi.2024.1435445 (PMC11341472; doi:10.3389/fnagi.2024.1435445)
Supplement: Supplementary file 1 [file Data_Sheet_1.docx]

**Supplementary Information**

**Modeling APOE ε4 Familial Alzheimer’s Disease in Directly Converted 3D Brain Organoids**

Yunkyung Kim^1#^, Hongwon Kim^1,2#^, Byounggook Cho^1#^, Saemin An^1^, Soi Kang^1^, Sumin Kim^1^ and Jongpil Kim^1*^

* Corresponding author: JONGPIL KIM: [jpkim153@dongguk.edu](mailto:jpkim153@dongguk.edu)

| **Sample ID** | **Genotype** | **Diagnosed** | **Age at sampling** | **Sex** | **Supplier** |
| --- | --- | --- | --- | --- | --- |
| GM23967 | APOE ε3/ε3 | - | 52 YR | Male | Coriell |
| AG21158 | APOE ε2/ε3 | - | 69 YR | Female | Coriell |
| AG04402 | APOE ε3/ε4 | Familial | 47 YR | Male | Coriell |
| AG05810 | APOE ε3/ε4 | Familial | 79 YR | Female | Coriell |
| AG11414 | APOE ε3/ε4 | Familial | 39 YR | Male | Coriell |

**Supplementary Table 1. List of human fibroblasts used for generating 3D induced brain organoids.**

**Supplementary Table 2. Top 100 DEGs in 3D APOE ε4 vs APOE ε3 organoids.**

|  | baseMean | log2FoldChange | lfcSE | stat | pvalue | padj |
| --- | --- | --- | --- | --- | --- | --- |
| PSG4 | 146.4707 | 10.58857 | 1.508102 | 7.021123 | 2.2E-12 | 8.65E-11 |
| TCF21 | 77.98814 | 9.679788 | 1.556291 | 6.219782 | 4.98E-10 | 1.49E-08 |
| NOP56P1 | 34.58808 | 8.505679 | 1.723172 | 4.93606 | 7.97E-07 | 1.44E-05 |
| ACAN | 29.3628 | 8.269512 | 1.749698 | 4.72625 | 2.29E-06 | 3.79E-05 |
| LOC105374809 | 24.57638 | 8.013109 | 1.783154 | 4.493784 | 7E-06 | 0.000104 |
| NLRP2 | 24.24147 | 7.992678 | 1.840782 | 4.342002 | 1.41E-05 | 0.000198 |
| PSG9 | 17.0921 | 7.489943 | 1.906322 | 3.929003 | 8.53E-05 | 0.000989 |
| PSCA | 16.71205 | 7.457199 | 1.91352 | 3.89711 | 9.73E-05 | 0.001111 |
| KRTAP1-5 | 160.0894 | 7.273262 | 0.834131 | 8.719566 | 2.79E-18 | 1.89E-16 |
| LOC728488 | 14.52548 | 7.255851 | 2.003661 | 3.621298 | 0.000293 | 0.003013 |
| HMSD | 12.69764 | 7.060684 | 2.042555 | 3.45679 | 0.000547 | 0.005199 |
| LOC284344 | 10.53068 | 6.791924 | 2.175205 | 3.122429 | 0.001794 | 0.014479 |
| LMO7-AS1 | 27.87818 | 6.744346 | 1.717769 | 3.926225 | 8.63E-05 | 0.000999 |
| IVL | 10.03899 | 6.721857 | 2.173854 | 3.092138 | 0.001987 | 0.015749 |
| TOX | 78.2959 | 6.654281 | 1.025484 | 6.488918 | 8.65E-11 | 2.86E-09 |
| LINC00484 | 9.468903 | 6.636717 | 2.232146 | 2.973245 | 0.002947 | 0.021767 |
| SPINK13 | 9.351313 | 6.619317 | 2.21975 | 2.98201 | 0.002864 | 0.021264 |
| LOC102724102 | 8.853668 | 6.54053 | 2.25453 | 2.901061 | 0.003719 | 0.026322 |
| IER3-AS1 | 8.840025 | 6.537088 | 2.322923 | 2.814165 | 0.00489 | 0.033144 |
| KRTAP9-9 | 8.794873 | 6.53127 | 2.259675 | 2.890358 | 0.003848 | 0.027066 |
| MTATP6P16 | 8.54605 | 6.489934 | 2.279929 | 2.846551 | 0.00442 | 0.030353 |
| LOC124909349 | 8.244388 | 6.436989 | 2.320919 | 2.773465 | 0.005546 | 0.036506 |
| HCG20 | 8.120843 | 6.417402 | 2.381602 | 2.694574 | 0.007048 | 0.044576 |
| LOC124902474 | 8.068003 | 6.406908 | 2.320371 | 2.761156 | 0.00576 | 0.037635 |
| LINC01436 | 65.1279 | 6.387413 | 1.053742 | 6.061645 | 1.35E-09 | 3.72E-08 |
| LOC124902309 | 16.0642 | 5.940922 | 1.895874 | 3.133606 | 0.001727 | 0.014061 |
| HCG17 | 15.89972 | 5.925306 | 1.948591 | 3.040816 | 0.002359 | 0.018127 |
| LINC00857 | 15.11406 | 5.85118 | 1.954193 | 2.994167 | 0.002752 | 0.020599 |
| LINC02154 | 268.6418 | 5.680875 | 0.465503 | 12.20374 | 2.97E-34 | 5.36E-32 |
| CYP21A1P | 17.36773 | 5.048909 | 1.598836 | 3.157865 | 0.001589 | 0.01312 |
| HLA-H | 787.3899 | 4.96084 | 1.028444 | 4.823636 | 1.41E-06 | 2.44E-05 |
| MTCO2P12 | 1943.648 | 4.83987 | 0.191223 | 25.31007 | 2.5E-141 | 3.9E-138 |
| EFCAB13-DT | 13.30817 | 4.655634 | 1.725059 | 2.698826 | 0.006958 | 0.044065 |
| TP53TG3D | 19.97185 | 4.619725 | 1.401591 | 3.296057 | 0.000981 | 0.008659 |
| LOC105379031 | 18.82209 | 4.568036 | 1.465835 | 3.116338 | 0.001831 | 0.014706 |
| SFRP4 | 54.34402 | 4.466879 | 0.848704 | 5.263178 | 1.42E-07 | 2.87E-06 |
| LINC01260 | 164.3573 | 4.400645 | 0.501756 | 8.770489 | 1.78E-18 | 1.22E-16 |
| SLC47A2 | 107.4575 | 4.388287 | 0.583772 | 7.51713 | 5.6E-14 | 2.58E-12 |
| NPTX1 | 1006.621 | 4.356817 | 0.212431 | 20.5093 | 1.78E-93 | 1.58E-90 |
| MTCO3P12 | 1840.714 | 4.273225 | 0.180041 | 23.73467 | 1.6E-124 | 2E-121 |
| TOX-DT | 24.80873 | 4.217631 | 1.266929 | 3.329018 | 0.000872 | 0.007836 |
| TRPM8 | 64.93674 | 4.177213 | 0.783851 | 5.329088 | 9.87E-08 | 2.04E-06 |
| EFNA5 | 326.6631 | 4.151143 | 0.349807 | 11.86696 | 1.76E-32 | 2.87E-30 |
| NAT8L | 71.54136 | 4.139985 | 0.712803 | 5.808034 | 6.32E-09 | 1.59E-07 |
| LOC105370185 | 31.42425 | 4.067423 | 1.139524 | 3.569404 | 0.000358 | 0.00358 |
| ERAP2 | 1111.639 | 4.05782 | 0.192301 | 21.10138 | 7.7E-99 | 7.5E-96 |
| MIR646HG | 23.28443 | 4.054895 | 1.307837 | 3.100458 | 0.001932 | 0.015415 |
| SMILR | 31.1387 | 4.022573 | 1.033327 | 3.892836 | 9.91E-05 | 0.001129 |
| GPRC5A | 133.9561 | 4.019956 | 0.507033 | 7.928389 | 2.22E-15 | 1.14E-13 |
| GPRC5D-AS1 | 43.51637 | 3.985291 | 0.878221 | 4.537911 | 5.68E-06 | 8.61E-05 |
| PKD2L2-DT | 17.17398 | -7.61125 | 1.909578 | -3.98583 | 6.72E-05 | 0.000807 |
| RASIP1 | 17.52427 | -7.63925 | 1.91128 | -3.99693 | 6.42E-05 | 0.000774 |
| TAGLN3 | 18.32792 | -7.70389 | 1.896636 | -4.06187 | 4.87E-05 | 0.000609 |
| CDC27P10 | 397.1348 | -7.70473 | 2.089981 | -3.68651 | 0.000227 | 0.002396 |
| CXCL5 | 18.49497 | -7.71804 | 1.876996 | -4.11191 | 3.92E-05 | 0.000504 |
| MCF2L | 21.05342 | -7.90429 | 1.83009 | -4.31907 | 1.57E-05 | 0.000218 |
| PAIP1P1 | 22.66185 | -8.01163 | 1.825485 | -4.38877 | 1.14E-05 | 0.000163 |
| PI16 | 64.76691 | -8.08099 | 1.561522 | -5.17507 | 2.28E-07 | 4.53E-06 |
| LXN | 24.18564 | -8.10497 | 1.782481 | -4.54701 | 5.44E-06 | 8.28E-05 |
| GBP6 | 24.67476 | -8.13331 | 1.7777 | -4.57519 | 4.76E-06 | 7.33E-05 |
| IL18 | 24.65745 | -8.13346 | 1.805731 | -4.50425 | 6.66E-06 | 9.95E-05 |
| NKAPL | 24.97952 | -8.15092 | 1.776657 | -4.58779 | 4.48E-06 | 6.93E-05 |
| LHX8 | 25.85804 | -8.2017 | 1.76995 | -4.63386 | 3.59E-06 | 5.7E-05 |
| CR1 | 26.06084 | -8.21254 | 1.758467 | -4.67028 | 3.01E-06 | 4.86E-05 |
| KRBOX1 | 26.93824 | -8.26009 | 1.749601 | -4.72113 | 2.35E-06 | 3.88E-05 |
| LRRC61 | 26.99355 | -8.26293 | 1.750687 | -4.71982 | 2.36E-06 | 3.9E-05 |
| XKR9 | 27.66815 | -8.29876 | 1.741479 | -4.76535 | 1.89E-06 | 3.2E-05 |
| UNC5D | 27.83295 | -8.30626 | 1.825704 | -4.54962 | 5.37E-06 | 8.2E-05 |
| ANGPT2 | 29.31121 | -8.38101 | 1.784783 | -4.69581 | 2.66E-06 | 4.34E-05 |
| DSC1 | 30.84477 | -8.45464 | 1.76244 | -4.79712 | 1.61E-06 | 2.75E-05 |
| CLEC2A | 31.00429 | -8.4639 | 1.744171 | -4.85268 | 1.22E-06 | 2.13E-05 |
| PTPN20 | 31.48363 | -8.48525 | 1.707843 | -4.9684 | 6.75E-07 | 1.23E-05 |
| LOC105379052 | 31.64955 | -8.49253 | 1.708395 | -4.97106 | 6.66E-07 | 1.22E-05 |
| GSDMA | 31.85347 | -8.50236 | 1.708587 | -4.97625 | 6.48E-07 | 1.19E-05 |
| TCN1 | 32.36968 | -8.52466 | 1.713535 | -4.9749 | 6.53E-07 | 1.2E-05 |
| DLK1 | 33.13759 | -8.55938 | 1.699148 | -5.03745 | 4.72E-07 | 8.92E-06 |
| LOC105369203 | 33.86638 | -8.58997 | 1.698467 | -5.05748 | 4.25E-07 | 8.07E-06 |
| KL | 35.94549 | -8.67634 | 1.677028 | -5.17364 | 2.3E-07 | 4.55E-06 |
| PEAR1 | 210.6631 | -8.78246 | 1.081244 | -8.12255 | 4.57E-16 | 2.53E-14 |
| LOC105369812 | 38.73496 | -8.78387 | 1.665758 | -5.27319 | 1.34E-07 | 2.73E-06 |
| HOXD13 | 41.76634 | -8.89338 | 1.654217 | -5.37619 | 7.61E-08 | 1.6E-06 |
| ILDR2 | 42.54178 | -8.91958 | 1.643123 | -5.42843 | 5.69E-08 | 1.22E-06 |
| CHRDL2 | 43.45605 | -8.95007 | 1.638887 | -5.46107 | 4.73E-08 | 1.03E-06 |
| CRABP1 | 46.74442 | -9.05508 | 1.628981 | -5.55874 | 2.72E-08 | 6.22E-07 |
| AIF1 | 46.78129 | -9.05618 | 1.62989 | -5.55631 | 2.76E-08 | 6.29E-07 |
| LRFN5-DT | 47.09695 | -9.06673 | 1.634754 | -5.54623 | 2.92E-08 | 6.62E-07 |
| CHST13 | 47.84305 | -9.08827 | 1.638534 | -5.54658 | 2.91E-08 | 6.61E-07 |
| ECEL1 | 54.2561 | -9.27064 | 1.604321 | -5.77854 | 7.54E-09 | 1.88E-07 |
| TLL1 | 56.71259 | -9.33412 | 1.595769 | -5.84929 | 4.94E-09 | 1.25E-07 |
| NAALADL1 | 61.86862 | -9.46015 | 1.587918 | -5.95758 | 2.56E-09 | 6.81E-08 |
| CHRNA9 | 62.57031 | -9.47625 | 1.582856 | -5.98681 | 2.14E-09 | 5.74E-08 |
| CSTA | 63.29044 | -9.49258 | 1.580001 | -6.00795 | 1.88E-09 | 5.1E-08 |
| TDRD9 | 69.74678 | -9.63231 | 1.574517 | -6.11763 | 9.5E-10 | 2.69E-08 |
| NHIP | 70.88341 | -9.65575 | 1.569143 | -6.15352 | 7.58E-10 | 2.2E-08 |
| FMO1 | 71.83455 | -9.67486 | 1.571201 | -6.15762 | 7.38E-10 | 2.15E-08 |
| SLC7A2 | 209.8157 | -9.77891 | 1.473005 | -6.63875 | 3.16E-11 | 1.1E-09 |
| CECR7 | 105.6836 | -10.2321 | 1.527897 | -6.69686 | 2.13E-11 | 7.58E-10 |
| COMP | 119.5041 | -10.4095 | 1.517892 | -6.85787 | 6.99E-12 | 2.61E-10 |
| PSPHP1 | 163.8865 | -10.8655 | 1.502367 | -7.23226 | 4.75E-13 | 1.97E-11 |
| MMP13 | 1279.377 | -11.3891 | 1.047527 | -10.8724 | 1.56E-27 | 1.9E-25 |


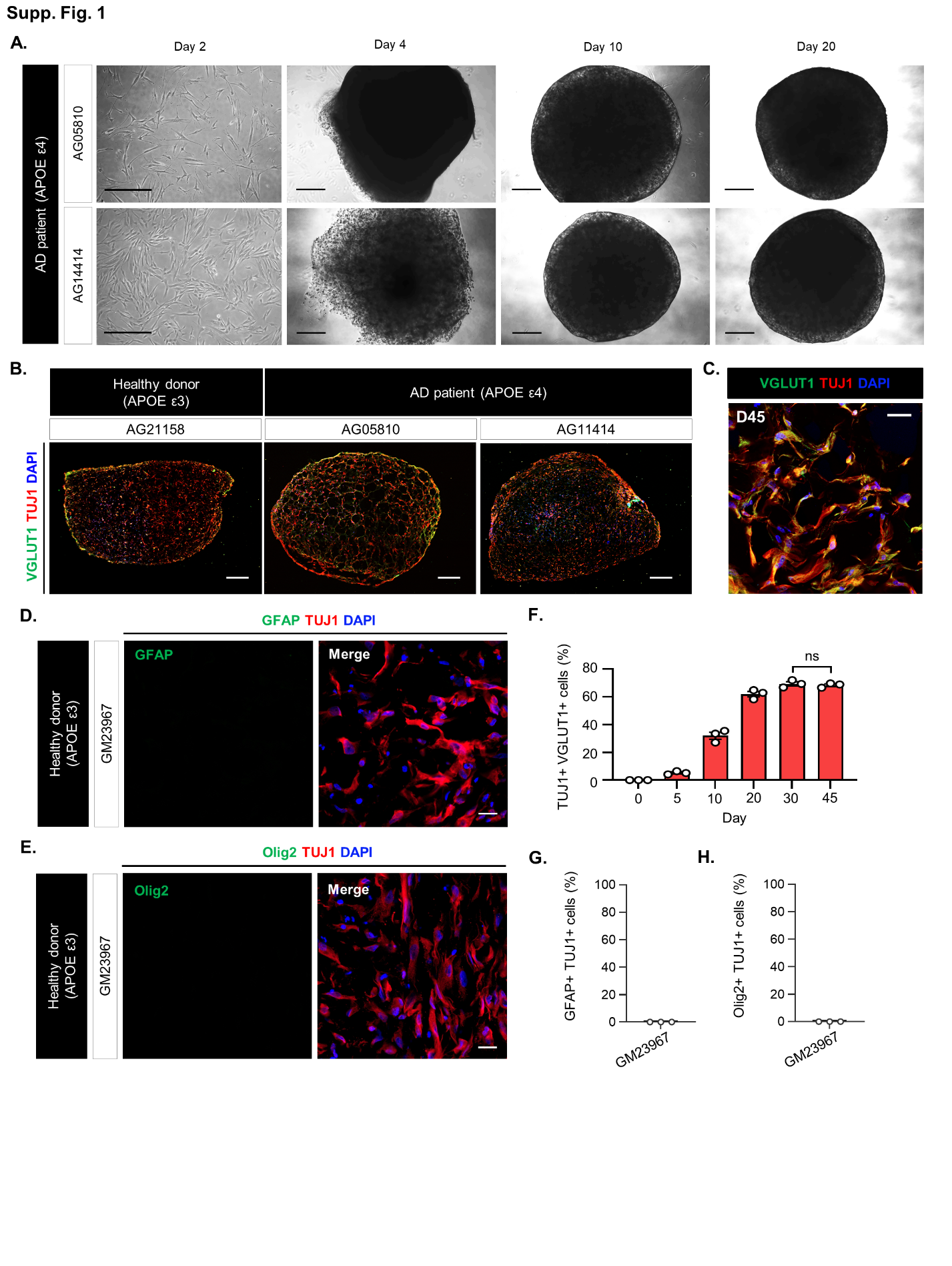


**Supplementary Figure 1.**

(A) Bright-field images of 2D-cultured human fibroblasts at day 2 and 3D-cultured induced brain organoids at day 4, 10, and 20 from AD patients (AG05810 and AG11414). Scale bar = 500 μm. (B) Immunostaining of Tuj1+Vglut1+ cells in induced brain organoids from a healthy donor (AG21158) or AD patients (AG05810 and AG11414) at day 20. Scale bar = 250 μm. (C) Immunostaining of Tuj1+Vglut1+ cells in 3D induced brain organoids derived from GM23967 at day 45. Scale bar = 40 μm. (D) Representative immunofluorescence images of Gfap+Tuj1+ cells in 3D induced brain organoids derived from GM23967. Scale bar = 20 μm. (E) Representative immunofluorescence images of Olig2+Tuj1+ cells in 3D induced brain organoids derived from GM23967. Scale bar = 20 μm. (F) Quantification of Tuj1- and Vglut1-positive cells in 3D cultured induced brain organoids at different time points. Data from day 0 to day 30 are reorganized from existing data in Figure 1F. Data represent mean ± SEM. Two-way ANOVA with Tukey’s multiple comparisons test, n = 3 per fibroblast. (G-H) Quantification of Tuj1+Gfap+ cells (G) or Tuj1+Olig2+ cells (H) in 3D induced brain organoids derived from GM23967. n = 3 organoids per fibroblast


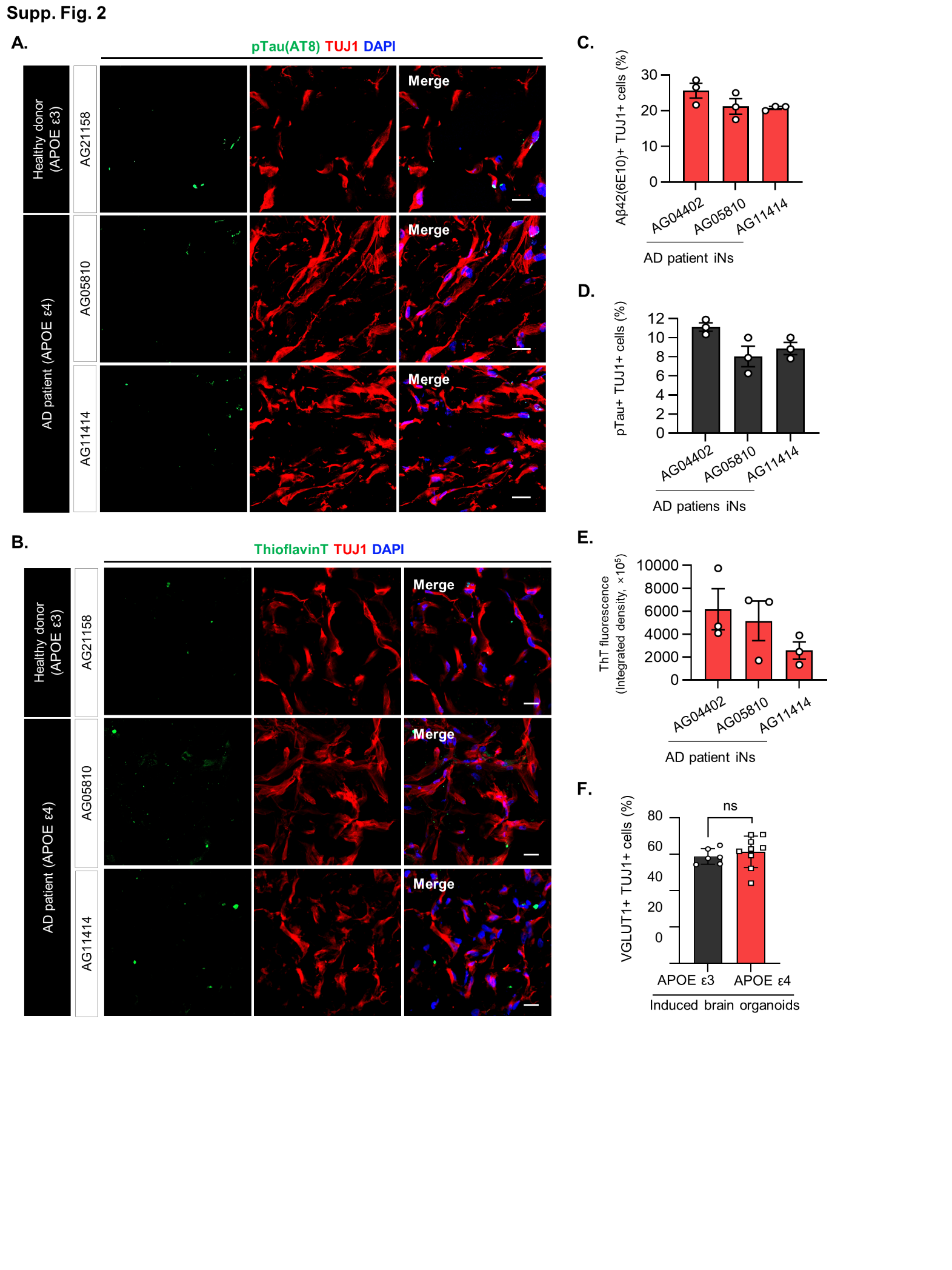


**Supplementary Figure 2.**

(A) Representative immunofluorescence images of phosphorylated tau (pTau) in 3D induced brain organoids from a healthy donor or AD patients at day 20. Scale bar = 20 μm. (B) Representative images of Thioflavin T (ThT) staining in 3D induced brain organoids from a healthy donor or AD patients at day 20. Scale bar = 20 μm. (C-D) Quantification of Aβ42(6E10)- and Tuj1-positive cells (C) and pTau- and Tuj1-positive cells (D) in 3D induced brain organoids derived from APOE ε4-fibroblasts. Data represent mean ± SEM. n = 3 per fibroblast; AG04402, AG05810, and AG11414. (E) Measurement of ThT fluorescence as integrated density values using ImageJ software in 3D induced brain organoids from APOE ε4 fibroblasts. Data represent mean ± SEM. n = 3 per fibroblast; AG04402, AG05810, and AG11414. (C), (D), and (E) are reorganized from existing data originally presented in Figures 2H, 2J, and 2K to display details separately for each patient-derived cell line. (F) Quantification of Tuj1- and Vglut1-positive cells in 3D induced brain organoids derived from APOE ε3 or APOE ε4 fibroblasts. Data represent mean ± SEM. Unpaired t test, n = 3 per fibroblast for both APOE ε3 (GM23967 and AG21158) and APOE ε4 (AG04402, AG05810, and AG11414). (F) is reorganized from data originally presented in Figures 1F and 2C.


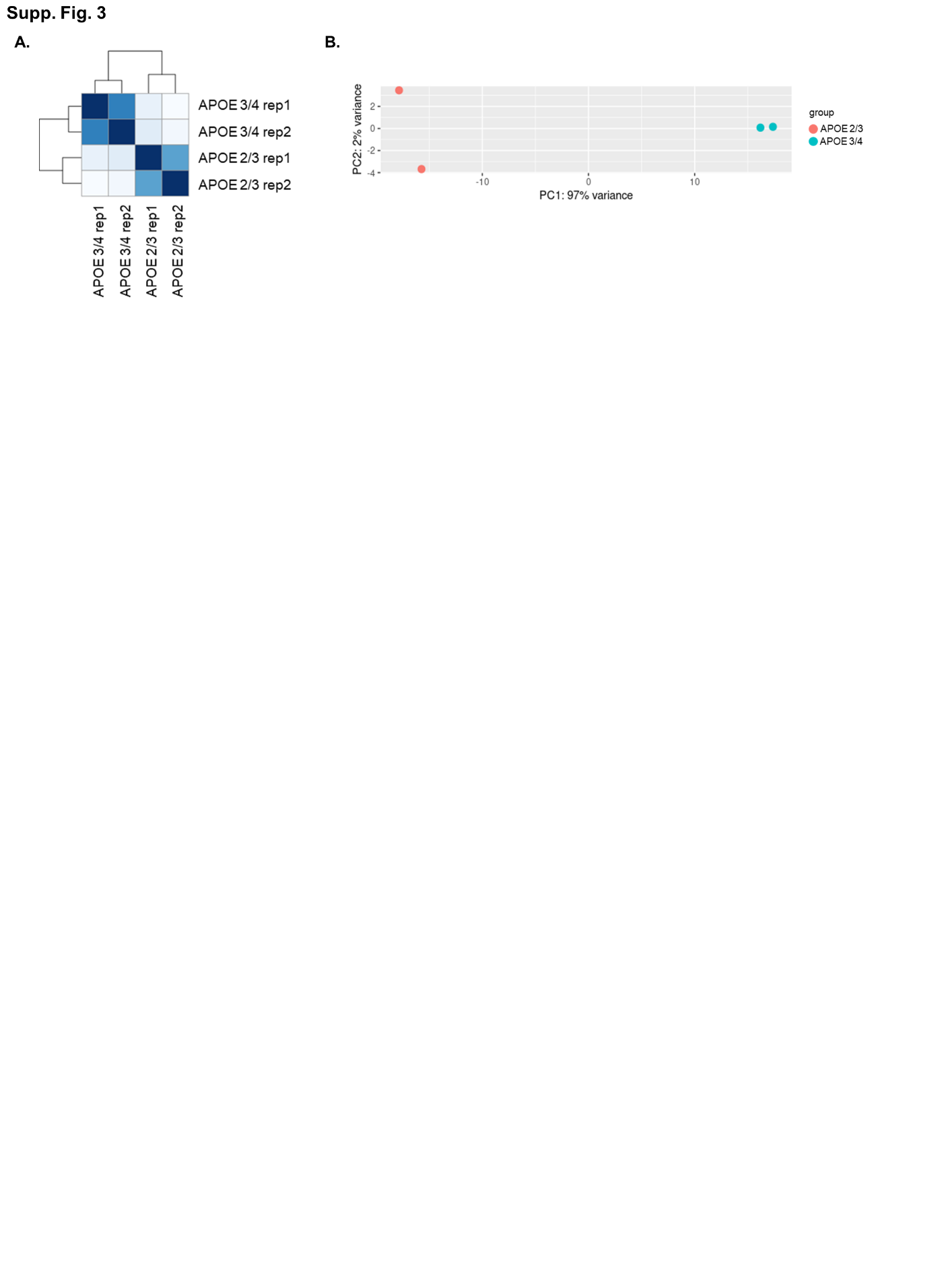


**Supplementary Figure 3.**

(A) The Pearson correlation coefficient between APOE ε4- and APOE ε3-induced brain organoids. Results showed that correlation coefficient of 2 pairs from APOE ε4 and APOE ε3 organoids. (B) Principal component analysis (PCA) of expression for two groups (APOE ε4 and APOE ε3). PC1, first principle component; PC2, second principle component.

**
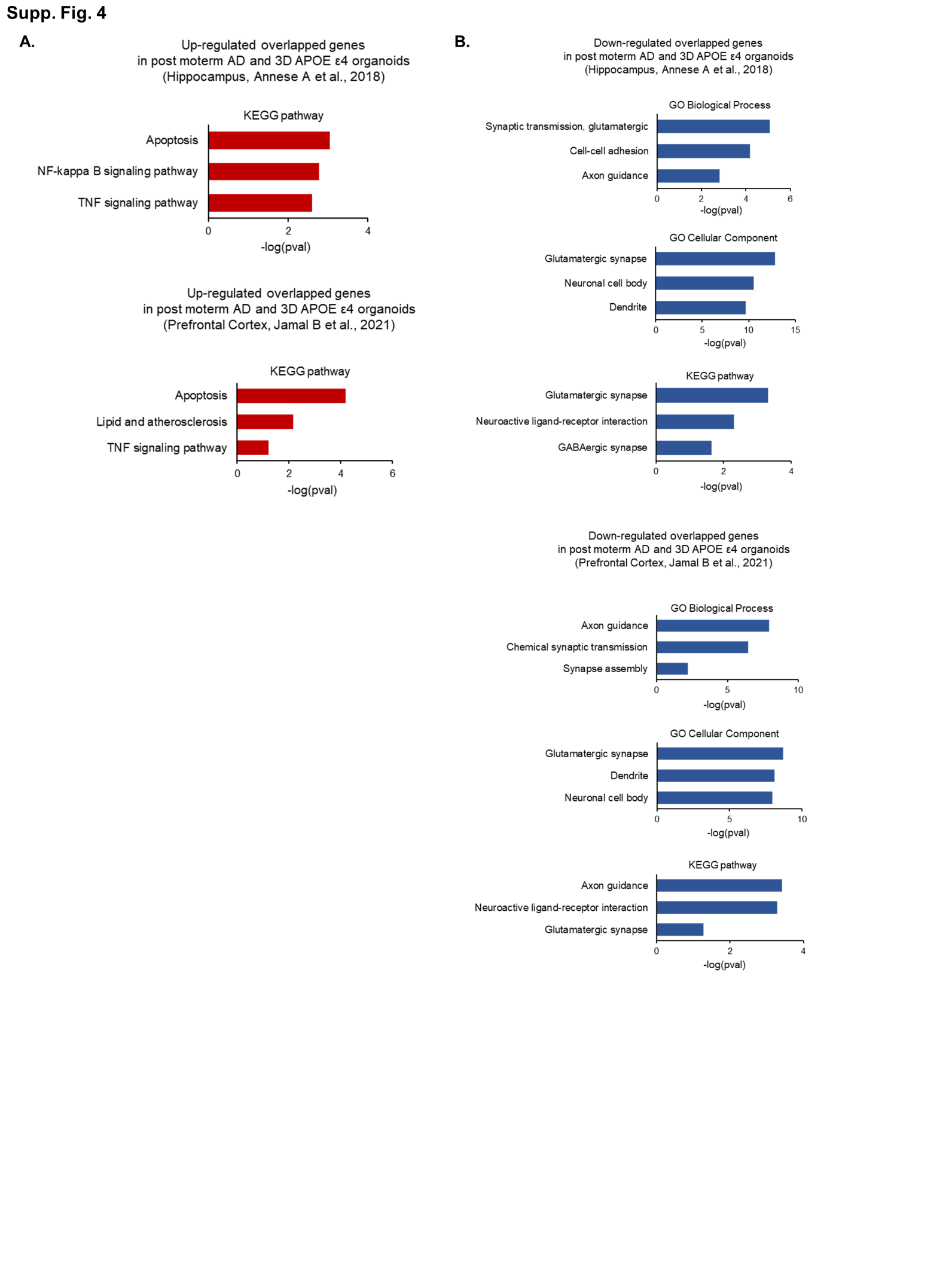
**

**Supplementary Figure 4.**

(A) Bar graph showing KEGG pathway from overlapping genes that are commonly up-regulated in APOE ε4-induced brain organoids and post-mortem brain (hippocampus and prefrontal cortex). (B) Bar graph showing GO biological process, cellular component and KEGG pathway from overlapping genes that are commonly down-regulated in APOE ε4-induced brain organoids and post-mortem brain (hippocampus and prefrontal cortex).


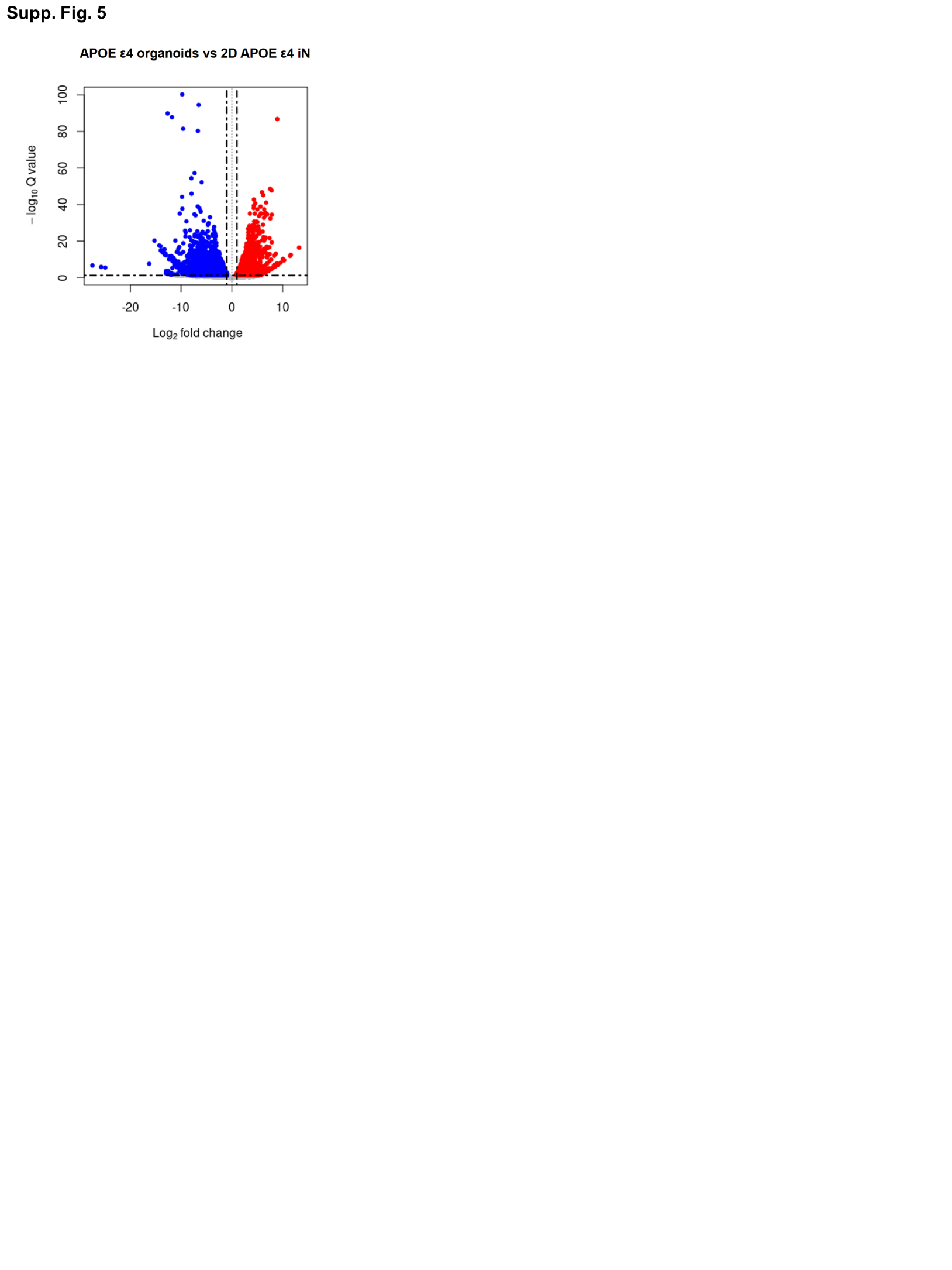


**Supplementary Figure 5.**

Volcano plot for DE genes of APOE ε4 induced brain organoids versus APOE ε4 2D induced neurons.
